# MicroRNA expression profile and identification of novel microRNA biomarkers for metabolic syndrome

**DOI:** 10.1080/21655979.2021.1952817

**Published:** 2021-07-16

**Authors:** Guanzhi Liu, Yutian Lei, Sen Luo, Zhuo Huang, Chen Chen, Kunzheng Wang, Pei Yang, Xin Huang

**Affiliations:** a Bone and Joint Surgery Center, Second Affiliated Hospital of Xi’an Jiaotong University, Xi’an, China; b Department of Cardiovascular Medicine, First Affiliated Hospital of Xi’an Jiaotong University, Xi’an, China

**Keywords:** Metabolic syndrome, miRNA, biomarker, bioinformatics, high-throughput sequencing

## Abstract

The lack of efficient biomarkers is the main reason for the inaccurate early diagnosis and poor treatment outcomes of patients with metabolic syndrome (MetS). The current study aimed to identify several novel microRNA (miRNA) biomarkers for metabolic syndrome via high-throughput sequencing and comprehensive bioinformatics analysis. Through high-throughput sequencing and differentially expressed miRNA (DEM) analysis, we first identified two upregulated and 36 downregulated DEMs in the plasma samples of patients with MetS compared to the healthy volunteers. Additionally, we also predicted 379 potential target genes and subsequently carried out enrichment analysis and protein–protein interaction network analysis to investigate the signaling pathways and functions of the identified DEMs as well as the interactions between their target genes. Furthermore, we selected two upregulated and top 10 downregulated DEMs with the highest |log2FC| values as the key microRNAs, which may serve as potential biomarkers for MetS. RT-qPCR was performed to validated these result. Finally, hsa-miR-526b-5p, hsa-miR-6516-5p was identified as the novel biomarkers for MetS.

## Graphical abstract

**Figure:**
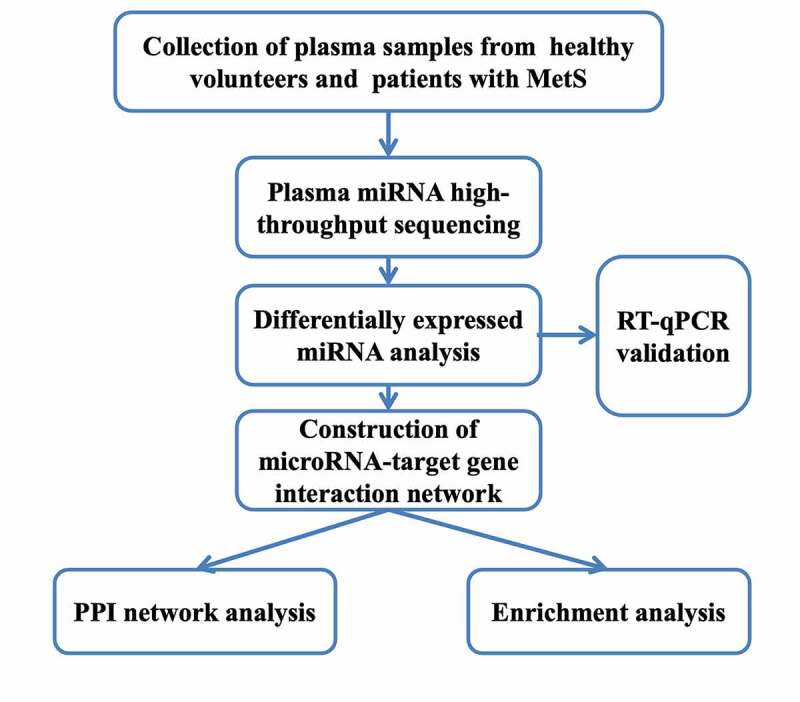

## 1. Introduction

Metabolic syndrome (MetS) is a common type of metabolic disorder which has several components including obesity, diabetes, insulin resistance, hypertension, and hyperlipidemia [1,2]. The development of metabolic syndrome and its associated components can cause negative cardiovascular outcomes [3]. However, the complex diagnostic approach limits the diagnosis of the disease, while the unclear mechanism of its pathogenesis affect the efficient treatment of MetS and MetS-related diseases in the early phase [4,5]. Ryo et al [6]. reported that plasma adiponectin could serve as a biomarker to be useful for metabolic syndrome management. Esteghamati et al [7]. demonstrated that leptin plays an independent role in development of MetS as a Serum biomarker. However, there is an urgent need for the identification of more accurate and more efficient biomarkers for MetS, which may aid in the development of novel strategies for the screening and diagnosis of MetS and provide novel insights into the underlying molecular mechanisms of MetS.

MicroRNAs (miRNAs) are a cluster of small non-coding RNAs containing 19–22 nucleotides [4]. They can bind to the 3′-untranslated regions (3′-UTRs) of their target genes and consequently repress the expression of these genes at the post-transcriptional level [8]. miRNAs are believed to play significant roles in several physiological processes of various diseases, such as cancers, cardiovascular diseases, and diabetes [9–11]. Previous studies have identified some miRNAs that are associated with MetS. Willeit et al. [12] reported that miRNA-122 is correlated with the onset of MetS and diabetes. Guo et al. [13] found that the expression levels of miR-122-5p, miR-21-5p, and miR-146a-5p were significantly upregulated in the subjects with MetS. High-throughput experimental techniques, such as microarray and high-throughput sequencing, are important approaches used for obtaining the microRNA expression data. Raitoharju et al. [14] analyzed and compared the whole blood miRNA expression profiles of patients with MetS with those of the healthy volunteers using microarray and selected nine MetS-associated miRNAs. However, little is known about the circulating miRNA biomarkers for MetS in plasma, which may act as potential diagnostic biomarkers for MetS.

To the best of our knowledge, this study is the first to analyze and compare the miRNA expression levels in the plasma samples of patients with MetS with those in the healthy volunteers via high-throughput sequencing based on the integrative bioinformatics approaches. We identified several novel miRNA biomarkers for MetS and performed RT-qPCR validattion. Finally, hsa-miR-526b-5p, hsa-miR-6516-5p were selected as plasma biomarkers for MetS that may play significant roles in the early diagnosis and treatment of MetS.

## 2. Materials and methods

### 2.1. Collection of clinical plasma samples

The Ethics Committee of the First Affiliated Hospital of Xi’an Jiaotong University approved this study (approval number: XJTU1AF2019LSL-014). The plasma samples were obtained from nine healthy volunteers and thirteen patients with MetS from the First Affiliated Hospital of Xi’an Jiaotong University (Table S2).

### 2.2. RNA extraction and next-generation sequencing

TRIzol LS Reagent (Invitrogen) was used to extract the total RNA of plasma samples according to the manufacturer’s instructions. Then, based on NEBNext Multiplex Small RNA Library Prep Set for Illumina, we generated the sequencing libraries following the standardized protocols. Sequencing libraries was qualified by Agilent Bioanalyzer 2100 system (Agilent). At last, the high-throughput sequencing for five healthy volunteers and five patients with MetS was conducted through the Illumina NextSeq 500 sequencing platform (Illumina).

### 2.3. Identification of differentially expressed miRNAs (DEMs)

Cutadapt software [15] and Solexa CHASTITY program were used to control and filter the raw sequencing data and identify the trimmed reads. Adapter sequences and sequences of length < 15 were removed. Subsequently, we obtained the miRNA reference data (version 22) and human reference genome indexing (hg38) using the miRBase database (http://www.mirbase.org/) and bowtie software (http://bowtie-bio.sourceforge.net/index.shtml). Sequencing alignment was performed using miRdeep2 [16] and microRNA was detected. DEMs were identified using the R package ‘edgeR’ [17]. The thresholds values for DEMs were set as |log2FC| ≥ 1 and *P* < 0.05.

### 2.4. Establishment of the microRNA-target gene interaction network

In this study, the TargetScan (http://www.targetscan.org/) and miRDB (http://www.mirdb.org/) databases were used to predict the target genes of the key DEMs. Only the target genes predicted by these two databases were selected as the target genes of key DEMs. Construction and visualization of the miRNA-target gene interaction network were carried out using the Cytoscape software v.3.7.2.

### 2.5. Function and pathway enrichment analysis

Gene Ontology (GO) function enrichment analysis and Kyoto Encyclopedia of Genes and Genomes (KEGG) pathway enrichment analysis were performed by ‘clusterProfiler’ package in R software with the threshold as P value <0.05 [18].

### 2.6. Construction of the protein–protein interaction (PPI) network and network module analysis

The PPI score was calculated using the Search Tool for the Retrieval of Interacting Genes/Proteins (STRING) database v.11.0. The PPI scores > 0.4 were considered to be statistically significant. Cytoscape v.3.7.2 was used to establish and visualize the PPI network. Furthermore, we performed network module analysis using the Molecular Complex Detection (MCODE) program. The major parameters were set as: k-score = 2, max depth = 100, degree cutoff = 2, and node score cutoff = 0.2. Moreover, the PPI network modules with score ≥ 5 were selected as the significant modules and subjected to further Kyoto Encyclopedia of Genes and Genomes (KEGG) pathway and Gene Ontology (GO) function enrichment analyses using the ‘clusterProfiler’ package.

### 2.7. Real-time quantitative polymerase chain reaction (RT-qPCR) validation

Plasma total RNA of eight patients with MetS and four healthy volunteers was extracted using miRNeasy Serum/Plasma Kit (Qiagen). Then, reverse transcription was conducted using miScript II RT Kit (Qiagen) according to the manufacturer’s protocol. Finally, RT-qPCR was performed using miScript SYBR Green PCR Kit (Qiagen, containing the universal primer). Relative expression of miRNAs were determined via the delta-delta Ct method (2-(ΔΔCt) method) and RNU6 was used as a endogenous control for normalization. The sequences of specific primers are as follows: hsa-miR-526b-5p (Forward, 5#'-TCTCTTGAGGGAAGCACTTTCTGT-3#'), hsa-miR-6516-5p (Forward, 5#'-CTTTGCAGTAACAGGTGTGAGCA-3#'), hsa-miR-137-3p (Forward, 5#'- CGCGTTATTGCTTAAGAATACGCGTAG-3#'), hsa-miR-499a-5p (Forward, 5#'- CGCGTTAAGACTTGCAGTGATGTTT-3#'), RNU6 (Forward, 5#'- AGAGAAGATTAGCATGGCCCCT −3#').

## 3. Results

This study perform high-throughput sequencing and integrative bioinformatics approaches to obtain the circulating miRNA expression data of patients with MetS and compare them to those from the healthy volunteers. The target genes of DEMs were predicted and further enrichment analysis and PPI network analysis were performed. Finally, we conducted the RT-qPCR to validate the expression of DEMs and identified hsa-miR-526b-5p and hsa-miR-6516-5p were significantly downregulated in the plasma of MetS patients compared with control group subjects (*P* <0.05) which could serve as plasma biomarker for MetS.

### 3.1. Identification of DEMs

**Figure 1.**
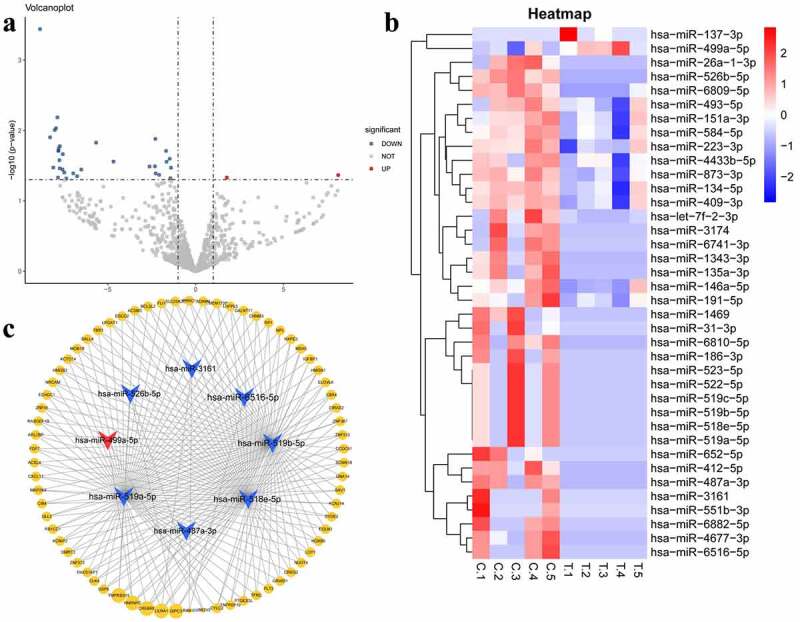
(a) Volcanoplot of the differentially expressed microRNAs (|log2FC| ≥ 1 and P value <0.05): red for up-regulated microRNAs and blue for down-regulated microRNAs. (b) Heatmap of the differentially expressed microRNAs (c) MicroRNA-target gene interaction network. Arrow nodes represent microRNAs and circle nodes represent target genes. Red for up-regulated microRNAs, blue for down-regulated microRNAs and yellow for target genes. The gradual spot size of target gene represents the number of microRNAs that can interact with it

In this study, 900 miRNAs were identified in the plasma samples from five patients with MetS and five healthy volunteers. miRNAs with |log2FC| values ≥ 1 and *P* < 0.05 were determined to be DEMs. The volcano plot and heatmap are shown in Figure 1(a,b). In this study, we selected two upregulated and the top 10 downregulated DEMs with the highest |log2FC| values as the key miRNAs. All DEMs expression data are shown in Table S1.

### 3.2. Establishment of the miRNA-target gene interaction network

We predicted the target genes of the top two upregulated (hsa-miR-137-3p, hsa-miR-499a-5p) and top 10 downregulated miRNAs (hsa-miR-526b-5p, hsa-miR-6516-5p, hsa-miR-551b-3p, hsa-miR-4677-3p, hsa-miR-487a-3p, hsa-miR-412-5p, hsa-miR-3161, hsa-miR-518e-5p, hsa-miR-519a-5p, and hsa-miR-519b-5p). Finally, we constructed a miRNA-target gene network, including eight miRNAs and 379 target genes as well as 522 miRNA-target gene interactions. Based on the cutoff criteria mentioned above, we eliminated hsa-miR-551b-3p, hsa-miR-4677-3p, hsa-miR-412-5p, and hsa-miR-137-3p from the analysis as they did not meet the outlined requirements. Only the target genes regulated by more than one miRNA are shown in Figure 1(c).

### 3.3. Pathway and function enrichment analyses

**Figure 2.**
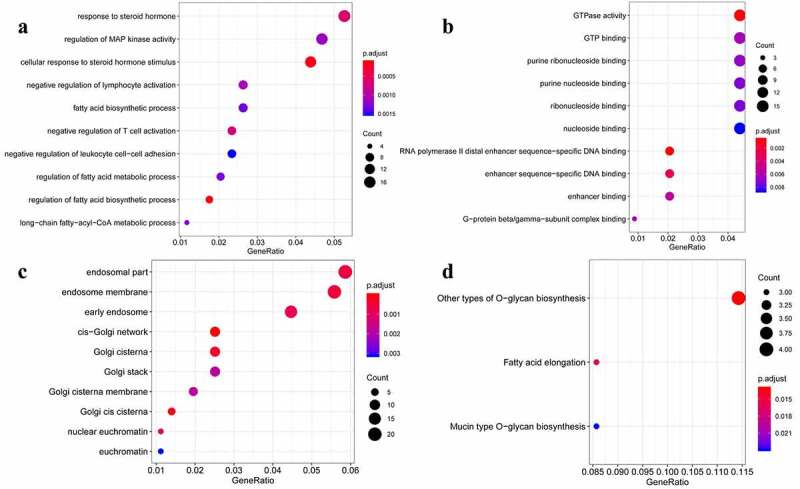
Gene Ontology (GO) function enrichment analysis and Kyoto Encyclopedia of Genes and Genomes (KEGG) pathway enrichment analysis. (a) Biological processes (BP). (b) Molecular function (MF). (c) Cellular component (CC). (d) KEGG signaling pathway

We performed the KEGG pathway and GO function enrichment analyses for the selected 379 target genes. GO function enrichment analysis revealed that the 379 target genes of the selected eight miRNAs were enriched in various biological processes, including the regulation of the activity of the mitogen-activated protein kinase (MAPK) as well as the response to steroid hormones. In addition, these target genes were significantly correlated with various molecular functions, such as the activity of the GTPase enzyme, GTP-binding activity, and cellular components, including the endosomal part, endosome membrane, and early endosome. Moreover, the KEGG pathway enrichment analysis indicated that these 379 target genes may be involved in other pathways, such as the O-glucan biosynthesis and fatty acid elongation pathways (Figure 2).

### 3.4. Construction of the PPI network

**Figure 3.**
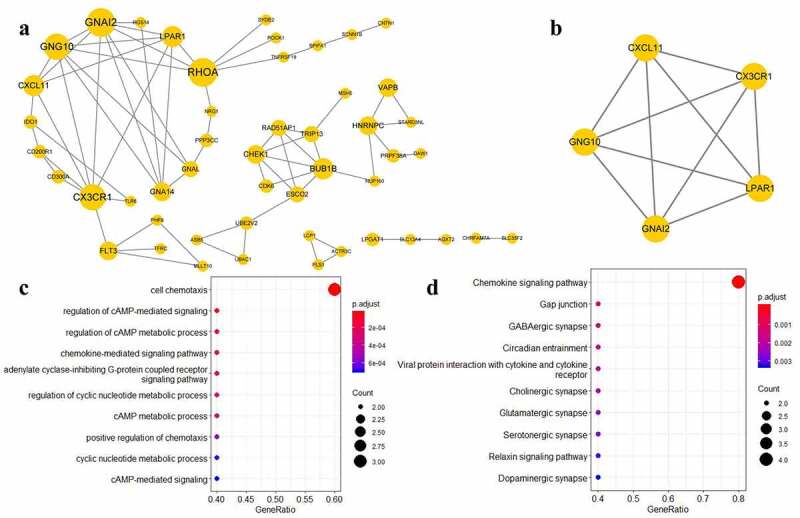
(a) Protein-protein interaction (PPI) network. (b) Key module of PPI network. (c,d) Kyoto Encyclopedia of Genes and Genomes pathway enrichment analysis and Gene Ontology function enrichment analysis

We conducted PPI analysis for these 379 target genes based on the STRING database. Based on the results of the analysis, we constructed a PPI network consisting of 110 target genes and only the genes with connectivity degree >1 were visualized (Figure 3(a)). Using this PPI network, we identified the target genes exhibiting high degrees of connectivity, such as the ras homolog family member A (RHOA), connectivity degree = 9), G protein subunit alpha i2 (*GNAI2*, connectivity degree = 9), G protein subunit gamma 10 (*GNG10*, connectivity degree = 8), and the C-X3-C motif chemokine receptor 1 (*CX3CR1*, connectivity degree = 8). These results indicate that the identified target genes exhibiting high degrees of connectivity may play important roles in the development of MetS.

### 3.5. PPI network module analysis

PPI network module analysis was performed to identify the significant subnets in the PPI network. Finally, we selected one module with an MCODE score ≥ 5, which included five target genes (Figure 3(b)): *GNAI2, GNG10, CX3CR1*, C-X-C motif chemokine ligand 11 (*CXCL11*), and the lysophosphatidic acid receptor 1 (*LPAR1*). Considering that this PPI module may be highly associated with MetS, further enrichment analysis was performed. The results of the GO function and KEGG pathway enrichment analyses showed that this PPI module may be correlated with the cell chemotaxis and chemokine signaling pathways (Figure 3(c,d)).

### 3.6. RT-qPCR validation

**Figure 4.**
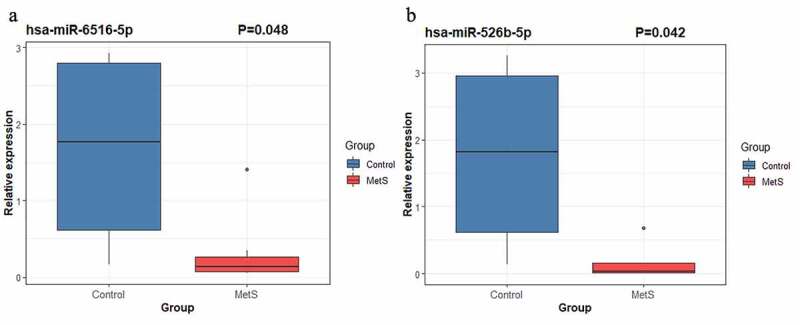
Relative expression levels of hsa-miR-526b-5p (*P* = 0.042) and hsa-miR-6516-5p (*P* = 0.048) in plasma of MetS patients compared with control group subjects detected by RT-qPCR

In order to validate these results and further identified MetS-associated plasma biomarkers, we carried out RT-qPCR to detect the expression level of four miRNA: hsa-miR-526b-5p, hsa-miR-6516-5p, hsa-miR-137-3p and hsa-miR-499a-5p. Our results showed hsa-miR-526b-5p (*P* =0.042) and hsa-miR-6516-5p (*P* =0.048) were significantly downregulated in the plasma of MetS patients compared with control group subjects (Figure 4). However, the differences expression of hsa-miR-137-3p (*P* =0.296) and hsa-miR-499a-5p (*P* =0.367) between MetS patients and healthy control subjects have no statistical significance (*P* <0.05). Hence, hsa-miR-526b-5p and hsa-miR-6516-5p could serve as plasma miRNA biomarkers for MetS.

## 4. Discussion

MetS is a complicated metabolic abnormality consisting of several components, such as obesity, diabetes, insulin resistance, hyperlipidemia, and hypertension [19,20]. Therefore, it is important to identify efficient biomarkers for MetS that can assist in the accurate early diagnosis and treatment of patients with MetS. To the best of our knowledge, our study is the first to perform high-throughput sequencing to obtain the circulating miRNA expression data from the plasma of the patients with MetS, which led to the identification of several potential plasma microRNA biomarkers for MetS.

First, we performed DEM analysis using the data obtained via high-throughput sequencing and identified two upregulated and 36 downregulated DEMs in the plasma samples of patients with MetS compared to the healthy volunteers. These miRNAs may be involved in the development of MetS. Previous studies have suggested that miR-137-3p may be associated with the production of insulin as well as the metabolism of lipids [21,22]. Ventriglia et al. demonstrated that the expression of miR-409-3p was downregulated in the plasma samples of diabetic mice [23]. Moreover, with the development of diabetes, there is a gradual decrease in the miR-409-3p levels in the plasma. The results of the present study support those from the previous studies and led to the identification of several novel circulating biomarkers for MetS, which may be crucial in determining the underlying molecular mechanism of MetS [24,25]. In addition, RT-qPCR was performed to validate the plasma expression of hsa-miR-526b-5p, hsa-miR-6516-5p, hsa-miR-137-3p and hsa-miR-499a-5p. Finally, we identified that hsa-miR-526b-5p and hsa-miR-6516-5p were significantly downregulated in the plasma of MetS patients compared with healthy volunteers (*P* <0.05); While the differences expression of hsa-miR-137-3p (*P* =0.296) and hsa-miR-499a-5p (*P* =0.367) between MetS patients and healthy volunteers have no statistical significance. These results suggested that these two novel microRNAs hsa-miR-526b-5p and hsa-miR-6516-5p can serve as plasma biomarkers for MetS.

Then, after predicting the target genes, we conducted GO function and KEGG signaling pathway enrichment analysis, which showed that these miRNAs were significantly enriched in various biological processes, such as the response to steroid hormones, regulation of MAPK activity, etc. Several studies have suggested the correlation between the increased activity of MAPK and MetS. Moreover, the MAPK pathway was found to play an important role in the multifactorial adverse cardiac remodeling associated with MetS [26]. Some studies have also reported that the activation of MAPK can enhance the occurrence of MetS components, such as insulin resistance and obesity [27,28]. Moreover, steroid hormone stimulus was found in both clinical and experimental studies and it has crucial effects on the progression of MetS [29,30]. Furthermore, the results of the cellular component enrichment analysis in our study indicated that these DEMs may be associated with cellular components, such as endosomal parts, endosome membranes, and early endosomes. The functions of exosomes in the glucose and lipid metabolism pathways have been broadly discussed in previous studies [31]. Recent evidence has shown that endosomal miRNAs can play a significant role in insulin resistance, obesity, and other MetS components [32,33].

Furthermore, we performed PPI network analysis and found several target genes exhibiting high degrees of connectivity, which may contribute to the development of MetS. RHOA belongs to the small GTPase family and the activation of the RhoA/Rho-kinase (ROCK) axis is reported to play important roles in various biological processes, such as proliferation and apoptosis [34]. Previous studies have detected high RhoA/ROCK activity in patients with MetS [35]. GNAI2 is known to serve as a crucial regulator of diet-induced obesity, which improves insulin sensitivity [36]. Meanwhile, CX3CR1 can bind to fractalkine (FKN; also known as CX3CL1) and modulate the secretion of insulin and atherosclerosis associated with insulin resistance [37,38]. Yin et al. conducted a clinical study and showed the correlation between the expression of circulating CX3CL1 and the development of MetS [39]. In our study, through PPI network module analysis, we selected a specific subnet associated with MetS from the whole PPI network, which consisted of five target genes: *GNAI2, GNG10, CX3CR1, CXCL11*, and *LPAR1*. The identification of this subnet further supported our initial assumption that the key target genes exhibiting high degrees of connectivity in the PPI network may play significant roles in the development of MetS.

## 5. Conclusion

In conclusion, the present study is the first to analyze the plasma miRNA expression profiles of patients with MetS and compare them to those from the healthy volunteers using next-generation sequencing and integrative bioinformatics approaches. Subsequently, we identifed several novel miRNA biomarkers that were highly associated with MetS. RT-qPCR was conducted to validate these results and finally we found that hsa-miR-526b-5p and hsa-miR-6516-5p were significantly downregulated in the plasma of MetS patients compared with healthy volunteers (*P* <0.05). These two novel plasma miRNA biomarkers may play significant roles in the early diagnosis and treatment of patients with MetS. Further functional experiments and mechanism experiments should be carried out focusing on these two miRNA.

## Supplementary Material

Supplemental Material

## Data Availability

The high-throughput sequencing data that supports the findings of this study is available from the corresponding author upon request.

## Research highlights

1. Performing plasma miRNA RNA-seq in MetS research for the first time.

2. identification of several novel plasma miRNAs highly associated with MetS.

3. hsa-miR-526b-5p and hsa-miR-6516-5p may serve as diagnosis biomarkers for MetS.
