# Supplementary material for: MicroRNA expression profile and identification of novel microRNA biomarkers for metabolic syndrome
Source: Bioengineered. 2021 Jul 16;12(1):3864–72. doi: 10.1080/21655979.2021.1952817 (PMC8806888; doi:10.1080/21655979.2021.1952817)
Supplement: Supplemental Material [file KBIE_A_1952817_SM0230.zip › supplementary/Table S1.docx]

| ID | log2FC | p_value |
| --- | --- | --- |
| hsa-miR-137-3p | 8.111237053 | 0.043035703 |
| hsa-miR-499a-5p | 1.774525529 | 0.046675433 |
| hsa-miR-191-5p | -1.404952836 | 0.033755055 |
| hsa-miR-151a-3p | -1.436654859 | 0.047638665 |
| hsa-miR-146a-5p | -1.477759663 | 0.025216967 |
| hsa-miR-223-3p | -1.653933802 | 0.027784367 |
| hsa-miR-409-3p | -1.685672757 | 0.019514839 |
| hsa-miR-134-5p | -2.077829691 | 0.042733703 |
| hsa-miR-493-5p | -2.27381817 | 0.040731952 |
| hsa-miR-584-5p | -2.295366953 | 0.013152029 |
| hsa-miR-4433b-5p | -2.31750282 | 0.032320965 |
| hsa-miR-873-3p | -2.627015074 | 0.032512446 |
| hsa-miR-6809-5p | -4.676913679 | 0.027661695 |
| hsa-let-7f-2-3p | -5.664030232 | 0.014887566 |
| hsa-miR-1343-3p | -6.509501016 | 0.03586008 |
| hsa-miR-135a-3p | -6.746584692 | 0.044607774 |
| hsa-miR-6810-5p | -6.96561742 | 0.040677826 |
| hsa-miR-186-3p | -7.363290677 | 0.048051119 |
| hsa-miR-6741-3p | -7.478360448 | 0.03946026 |
| hsa-miR-1469 | -7.560908886 | 0.021667845 |
| hsa-miR-6882-5p | -7.572270306 | 0.035831999 |
| hsa-miR-26a-1-3p | -7.729177648 | 0.016709709 |
| hsa-miR-31-3p | -7.731439449 | 0.034514517 |
| hsa-miR-652-5p | -7.755103599 | 0.02643354 |
| hsa-miR-3174 | -7.793027447 | 0.017936285 |
| hsa-miR-518e-5p | -7.807573709 | 0.019414642 |
| hsa-miR-519a-5p | -7.807573709 | 0.019414642 |
| hsa-miR-519b-5p | -7.807573709 | 0.019414642 |
| hsa-miR-519c-5p | -7.807573709 | 0.019414642 |
| hsa-miR-522-5p | -7.807573709 | 0.019414642 |
| hsa-miR-523-5p | -7.807573709 | 0.019414642 |
| hsa-miR-3161 | -7.836179241 | 0.046594255 |
| hsa-miR-412-5p | -7.866071705 | 0.006508063 |
| hsa-miR-487a-3p | -7.947486984 | 0.009294316 |
| hsa-miR-4677-3p | -8.019713124 | 0.009847617 |
| hsa-miR-551b-3p | -8.102059646 | 0.033614508 |
| hsa-miR-6516-5p | -8.284334015 | 0.012485321 |
| hsa-miR-526b-5p | -8.853893576 | 0.000362216 |
