# Supplementary material for: MicroRNA expression profile and identification of novel microRNA biomarkers for metabolic syndrome
Source: Bioengineered. 2021 Jul 16;12(1):3864–72. doi: 10.1080/21655979.2021.1952817 (PMC8806888; doi:10.1080/21655979.2021.1952817)
Supplement: Supplemental Material [file KBIE_A_1952817_SM0230.zip › supplementary/Table S2.docx]

| name | Sex | Age | MetS | Subjects |
| --- | --- | --- | --- | --- |
| Patient1 | M | 46 | Yes | RNA-seq |
| Patient2 | F | 47 | Yes | RNA-seq |
| Patient3 | F | 19 | Yes | RNA-seq |
| Patient4 | F | 33 | Yes | RNA-seq |
| Patient5 | M | 23 | Yes | RNA-seq |
| Control1 | F | 26 | No | RNA-seq |
| Control2 | M | 31 | No | RNA-seq |
| Control3 | M | 25 | No | RNA-seq |
| Control4 | F | 44 | No | RNA-seq |
| Control5 | M | 35 | No | RNA-seq |
| Patient6 | M | 59 | Yes | RT-qPCR |
| Patient7 | F | 32 | Yes | RT-qPCR |
| Patient8 | M | 44 | Yes | RT-qPCR |
| Patient9 | F | 61 | Yes | RT-qPCR |
| Patient10 | M | 44 | Yes | RT-qPCR |
| Patient11 | M | 40 | Yes | RT-qPCR |
| Patient12 | M | 34 | Yes | RT-qPCR |
| Patient13 | F | 23 | Yes | RT-qPCR |
| Control6 | F | 40 | No | RT-qPCR |
| Control7 | F | 20 | No | RT-qPCR |
| Control8 | M | 35 | No | RT-qPCR |
| Control9 | M | 44 | No | RT-qPCR |
